# Inhibition of the hepatic *Nlrp3* protects dopaminergic neurons via attenuating systemic inflammation in a MPTP/p mouse model of Parkinson’s disease

**DOI:** 10.1186/s12974-018-1236-z

**Published:** 2018-07-02

**Authors:** Chen Qiao, Qian Zhang, Qingling Jiang, Ting Zhang, Miaomiao Chen, Yi Fan, Jianhua Ding, Ming Lu, Gang Hu

**Affiliations:** 1 Jiangsu Key Laboratory of Neurodegeneration, Department of Pharmacology, Nanjing Medical University, 101 Longmian Avenue, Nanjing, 211166 Jiangsu China; 2 Department of Pharmacology, Nanjing University of Chinese Medicine, 138 Xianlin Avenue, Nanjing, 210023 Jiangsu China; 3 Neuroprotective Drug Discovery Key Laboratory, Department of Pharmacology, Nanjing Medical University, 101 Longmian Avenue, Nanjing, 211166 Jiangsu China; 4 Department of Clinical Pharmacy, Affiliated Hospital of Jiangsu University, Jiangsu University, Zhenjiang, 212001 Jiangsu China

**Keywords:** NLRP3 inflammasome, Systemic inflammation, Neurodegeneration, IL-1β, Parkinson’s disease

## Abstract

**Background:**

Parkinson’s disease (PD) is a neurodegenerative disorder with progressive loss of dopaminergic (DA) neurons. Systemic inflammation is shown to initiate and exacerbate DA neuronal degeneration in the substantia nigra. The infiltration and transformation of immune cells from the peripheral tissues are detected in and around the affected brain regions of PD patients. Our previous studies demonstrated the crucial role that microglial Nod-like receptor protein (NLRP) 3 inflammasome plays in the pathogenesis of PD. Nevertheless, the direct linkage between peripheral inflammation and DA neuron death remains obscure.

**Methods:**

In the present study, we detected the NLRP3 expressions in the midbrain, liver, and bone marrow-derived macrophages in response to 1-methyl-4-phenyl-1, 2, 3, 6-tetrahydropyridine (MPTP) acute and chronic challenge. We then used a tail vein injection of *Nlrp3*-siRNA wrapped with lentivirus to explore the potential influence of hepatic NLRP3 inflammasome-mediated inflammation on neuronal injury in a mouse model of PD via immunohistochemistry, ELISA, and Western blotting analysis.

**Results:**

We showed that si*Nlrp3* downregulated the NLRP3 protein expression and inhibited the activation of NLRP3 inflammasomes in mice livers. The tail vein injection of LV3-si*Nlrp3* reduced the liver pro-inflammatory cytokine production, which subsequently alleviated MPTP-triggered microglial activation and DA neuron loss in the midbrain. These findings indicated that inhibition of hepatic NLRP3 inflammasome weakens inflammatory cytokines spreading into the brain and delays the progress of neuroinflammation and DA neuronal degeneration.

**Conclusion:**

This study gives us an insight into the direct linkage between liver inflammation and DA neuron damage in the pathogenesis of PD and provides the potential target of NLRP3 for developing novel drugs for PD therapy.

**Electronic supplementary material:**

The online version of this article (10.1186/s12974-018-1236-z) contains supplementary material, which is available to authorized users.

## Background

Parkinson’s disease (PD) is a common age-related neurodegenerative disease that is induced by multitudinous environmental and inherited factors [1]. PD is characterized by the progress degeneration of dopaminergic (DA) neurons in the substantia nigra pars compacta (SNc), as well as clinical motor dysfunction [2]. The nigrostriatal DA neurons are vulnerable to multiple insults, including inflammatory attacks. Neuroinflammation and systemic inflammation are considered important contributors to the pathogenesis of PD [3, 4], where results of epidemiological studies show a reduced risk of PD with the use of anti-inflammatory medications, specifically non-steroidal anti-inflammatory drugs [5, 6]. The efficacy of anti-inflammatory drugs within the PD clinical treatment and compatibility with motor symptom controlling drugs requires further examination.

Most researches have focused on the neuroinflammation process of PD, while systemic inflammatory responses, especially peripheral inflammation acts as an inescapable risk factor during the pathological process of PD [7, 8]. The neurovascular function becomes altered in aging and neurodegenerative disorders, leading to abnormal states, such as increased blood brain barrier (BBB) permeability and failure of enzymatic function [9]. When inflammatory reactions occur, the infiltration of peripheral inflammatory cytokines and immune cells permeate through the BBB to induce the degeneration of dopaminergic neurons in the SNc [10]. The peripheral inflammation amplifies the inflammatory cascade in the central nervous system (CNS) and further exacerbates neurodegeneration [11]. The ability to reduce or control the systemic inflammatory response has important significance in developing novel anti-inflammatory medicines for PD therapy.

Previous reports show that the inflammatory cytokines, such as tumor necrosis factor-α (TNF-α), interleukin-1β (IL-1β), and interferon-γ (IFN-γ), have higher levels in the serum of PD patients [12]. IL-1β is a key pro-inflammatory cytokine throughout the pathological damage of PD [13], and our previous study had shown that the increased IL-1β levels were primarily produced by nod-like receptor protein 3 (NLRP3) inflammasome activation in the midbrain and the microglia of PD model [14]. The application of IL-1β receptor blockers to postpone the process of PD remains controversial in experimental animal studies, due to uncontrollable factors in regulating the production and maturation of IL-1β [15, 16]. The activation of inflammasome promotes the maturation and the release of several pro-inflammatory cytokines, such as IL-1β and IL-18. The activation of inflammasomes requires tight control to prevent excessive inflammation [17, 18]. Further research is needed to determine if the inflammasome could be a linking bridge in regulating systemic inflammation from the peripheral tissues to CNS, which will allow us to understand the exact events that lead to inflammation in PD.

The current study established acute 1-methyl-4-phenyl-1, 2, 3, 6-tetrahydropyridine (MPTP) and chronic MPTP/probenecid (MPTP/p)-induced PD model with C57BL/6J mice in order to determine the role that NLRP3 inflammasomes play in the liver and the brain involved during the pathogenesis of PD. We used siRNA wrapped with lentivirus (LV3-siNlrp3) to reduce the liver NLRP3 expression via tail vein injection. The siNlrp3 inhibited the MPTP-induced activation of NLRP3 inflammasome in the liver and pro-inflammatory cytokine release particularly in IL-1β. The LV3-siNlrp3 suppressed the activation and the proliferation of microglia and alleviated the loss of DA neurons in the SNc in MPTP-injected mice. Our findings suggested that hepatic NLRP3 could act as a potential target for the linkage of inflammatory response from the liver to the brain during the pathogenesis of PD.

## Methods

### Animals and treatments

Three to 4-month-old male C57BL/6J mice were purchased from Nanjing Medical University (Nanjing, Jiangsu, China) and maintained in light/dark (12-h light/12-h dark), temperature (22–24 °C), and humidity-controlled rooms. The mice were fed standard food with free access to drinking water. All experiments were performed in strict accordance with the National Institutes of Health Guide for the Care and Use of Laboratory Animals.

The mice were injected with the negative control siRNA wrapped with lentivirus (LV3-NC) or the *Nlrp3* siRNA wrapped with lentivirus (LV3-GFP-siNlrp3 or LV3-siNlrp3) (20 μl per mice, 10^9^ TU/ml) via the tail vein [19]. The siRNA wrapped with lentivirus was synthesized by Genepharma (Shanghai, China) for gene silencing of mouse *Nlrp3.* After 1 week, the mice were injected with MPTP (20-mg/kg body weight) administered four times at 2-h intervals via intraperitoneal and euthanized after either 90 min, 120 min, 240 min, or 7 days post injection (the total dose per mouse was 80-mg/kg body weight) [20] for acute MPTP PD model. For chronic MPTP/p PD model, the mice were injected subcutaneously with 20 mg/kg MPTP (Sigma, St. Louis, MO, USA) in saline and 1 h later intraperitoneally with 250 mg/kg probenecid in DMSO every 3.5 days over a period of 5 weeks. The mice were euthanized 7 days after the last injection. The control mice were treated with saline only [21]. The midbrains, livers, and serum samples were collected during these time periods. The siRNA sequences were (5′ to 3′): LV3-NC TTCTCCGAACGTGTCACGT and LV3-siNlrp3 GGTTCTGAGCTCCAACCATTC.

### Liver histology

The livers were prefixed by perfusion with 4% paraformaldehyde in 0.01 M phosphate buffer (PBS; PH 7.4, 4 °C) and dehydrated using graded 20% sucrose for 3 days and then 30% sucrose for 3 days. The liver tissues were then sectioned on Leica freezing microtomes at 10 μm using a freezing microtome. Liver biopsy sections (10 μm) were stained with hematoxylin and eosin (HE staining) and then evaluated by a double-blinded hepatopathologist or by directly observed green fluorescent protein (GFP) with a fluorescence microscope (Additional file 1).

### Analysis of monoamine oxidase B (MAO-B) activity

All enzymatic assays were performed in a phosphate buffer (pH 7.5) supplemented with 0.1% Triton X-100 at 37 °C. The activities of the recombinant MAO-B proteins were normalized with the ELISA kit (Senbeijia Biomart, Nanjing, Jiangsu, China), where *p*-tyramine hydrochloride was used as a substrate. The activity was assayed by monitoring the rate of resorufin formation at 560 nm, in accordance with the manufacturer’s guidelines. All measurements were performed in triplicate. Lysates from tissues were prepared from RIPA lysis buffer. The protein concentration was detected with a BCA assay kit (Beyotime Biotechnology, Nanjing, Jiangsu, China). All plates were read on a microplate reader (Thermo Fisher Scientific, USA) at 562 nm. The activity of MAO-B was analyzed in unit per milligram pre protein.

### Immunohistochemistry

The protocol for immunohistochemical staining was described in previous literature [21]. The brain was prefixed via perfusion with 4% paraformaldehyde in 0.01 M PBS (PH 7.4, 4 °C). The brain tissues were sectioned on a Leica freezing microtome at 30-μm sections through the midbrain (from approximately − 2.5 to − 3.88 mm from bregma, according to the whole mouse brain atlas) using a freezing microtome. Midbrain sections were washed three times with 0.01 M PBS, 3% H_2_O_2_ was added for 30 min to eliminate endogenous peroxidase, the sections were washed with PBS three times, and then the sections were incubated for 1 h in blocking a solution (0.3% Triton X-100 and 5% bovine serum albumin (BSA) in PBS), followed by incubation overnight with either primary antibody (anti-Tyrosine hydroxylase (TH) antibody (1:4000, Sigma) or anti-ionized calcium binding adaptor molecule 1 (IBA-1) antibody (1:1000, wako)) in order to detect DA neurons or microglia. The samples were then for 1 h with secondary antibodies. Immunoreactivity was visualized via incubation in DAB. Control staining was performed without the primary antibodies. The specimens were observed under Microbrightfield Stereo Investigator software (Microbrightfield, Williston, VT, USA) for visualization and photography.

### Enzyme-linked immunosorbent assay (ELISA)

Following euthanasia, the blood from mice was laid to rest 4 h at room temperature. The blood samples were centrifuged at 3000*g* for 10 min, and the serum was transferred for the ELISA test. The concentrations of IL-1β, caspase-1, TNF-α, IL-12, and IL-18 in serum were measured by the mouse ELISA Kits (R&D, USA), in accordance to the manufacturer’s instructions. The plates were read on a microplate reader (Thermo Fisher Scientific, USA) at 450 nm.

### High-performance liquid chromatography (HPLC) analysis of striatal methyl-phenyl-pyridinium (MPP^+^) concentration

The mice were euthanized 90, 120, or 240 min post MPTP injection. The striata were dissected, immediately frozen, and stored at − 80 °C until ready for analysis. On the day of the assay, the tissue samples were sonicated in 10 vol of 5% tricholoracetic acid containing 5 μg/ml of 4-phenylpyridine (Sigma, USA) as the internal standard. The samples were centrifuged at 3000*g* for 10 min and then 20 μl of the supernatant was injected onto a cation-exchange Ultracyl-CS column (Waters chromatographic system, Japan). The mobile phase consisted of 90% a solution with 0.1 M acetic acid and 75 mM triethylamine-HCl (pH 2.35 adjusted with formic acid), and 7% acetonitrile. The flow rate was 1.5 ml/min. An external calibration curve was used to express the final amount in the tissue sample as microgram per gram (μg/mg) wet tissue for MPP^+^.

### Cell culture for BMDMs

Bone marrow-derived macrophages (BMDMs) were derived from tibia and the femoral bone marrow cells and cultured for 7 days in Dulbecco’s modified essential media complemented with 10% fetal bovine serum, 1% penicillin/streptomycin (vol/vol), and 50 nM granulocyte-macrophage colony stimulating factor (GM-CSF). The purity of BMDM culture was > 95%, which was as determined with immunocytochemistry.

### Western blotting analysis

The midbrain, the liver, and the BMDM cells protein lysates were fractionated with a RIPA lysis buffer. The protein was electrophoresed through a 10–15% SDS-polyacrylamide gel and blotted through the PVDF-membrane. The membranes were probed with the following primary antibodies: rabbit anti-Caspase-1/pro-caspase-1 (1:500, Millipore, USA), mouse anti-IL-1β/pro-IL-1β (1:1000, Sigma, USA), mouse anti-NLRP3 (1:1000, Adipogen, USA), rabbit anti-NLRP1 (1:5000, Cell Signaling, USA), rabbit anti-NLRP2 (1:1000, abcam, USA), goat anti-NLRC4 (1:2000, Santa Cruze, USA), rabbit anti-AIM2 (1:5000, Santa Cruze, USA), and mouse-β-actin (1:1000, Sigma, USA). The blots were incubated secondary antibodies and the signals were detected by the enhanced chemiluminescence (ECL) (Pierce, Rockford, IL, USA). The membranes were analyzed using an Image Quant LAS 4000 Chemiluminescence Imaging System (GE Healthcare, USA).

### Statistical analysis

Data was initially examined for equal variance and then subjected to two-way repeated-measures ANOVA using time and treatments as variables, with Turkey’s post hoc tests at the treatment. Student’s *t* tests were used for single variant analyses. In all studies, *n* indicated the number of animals used in each group and a critical value of *p* < 0.05 was used. All values were reported as mean ± SEM.

## Results

### NLRP3 inflammasome plays a critical role in MPTP-induced mouse model of PD

**Fig. 1:**
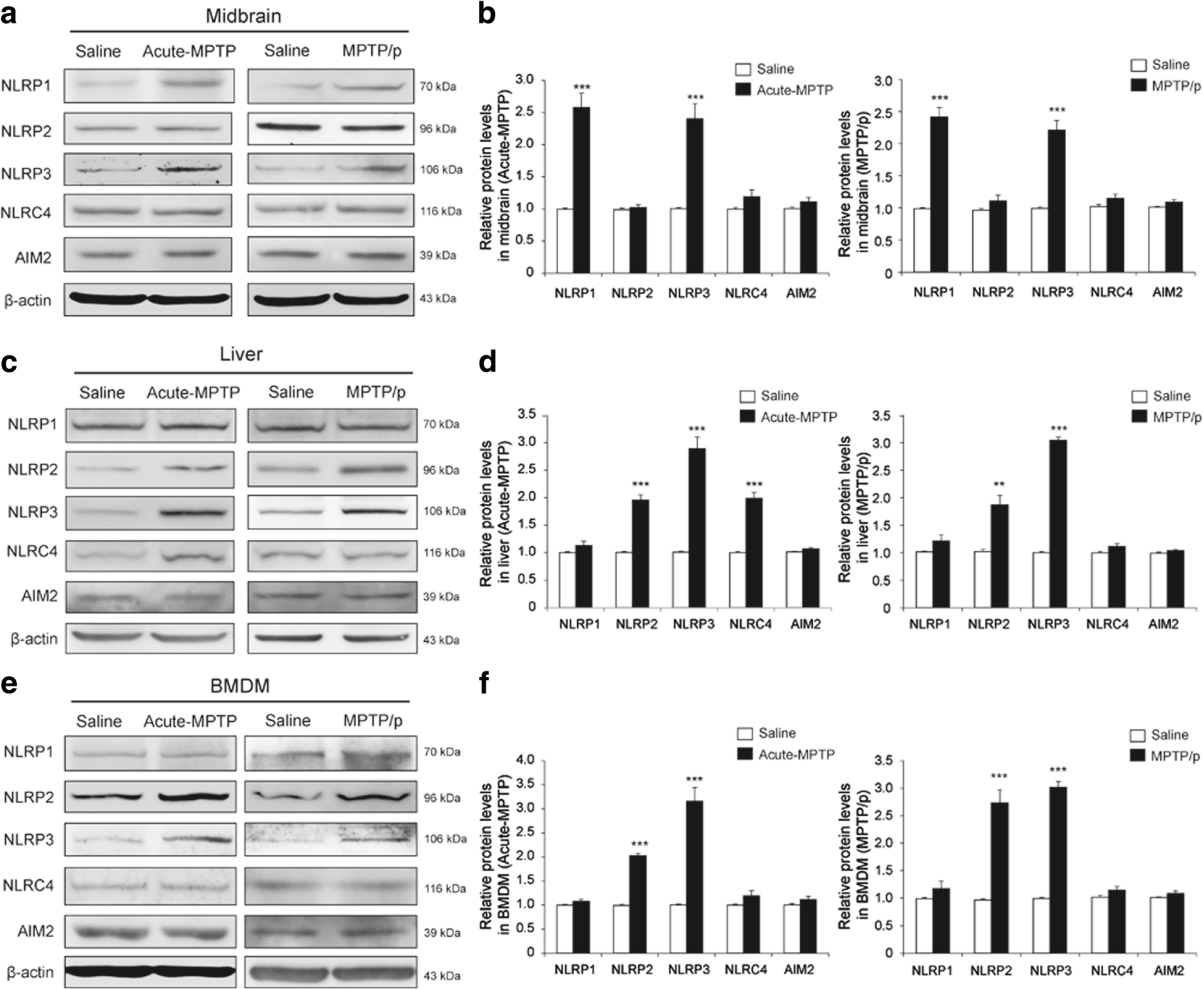
The expression of NLRP3 protein exhibited the most changes in the midbrain, the liver, and the BMDM of MPTP-induced mice. **a** The expression of NLRP1 and NLRP3 was not only higher in the midbrain of the acute MPTP model mice, but also in the MPTP/p model mice. **b** The analysis of NLRP1, NLRP2, NLRP3, NLRC4, and AIM2 expression in the midbrain of the acute MPTP model mice (left), as well as the analysis in the MPTP/p model mice (right). **c** The expressions of NLRP2, NLRP3, and NLRC4 were higher in the liver of the acute MPTP model mice. The expressions of NLRP2 and NLRP3 were higher compared to the saline group in the liver of MPTP/p model mice. **d** The analysis of NLRP1, NLRP2, NLRP3, NLRC4, and AIM2 expression in the liver of the acute MPTP model mice (left), as well as the analysis in the MPTP/p model mice (right). **e** The expression of NLRP2 and NLRP3 was not only higher in the BMDM of the acute MPTP model mice, but also in the MPTP/p model mice. **f** The analysis of NLRP1, NLRP2, NLRP3, NLRC4, and AIM2 expression in the BMDM of the acute MPTP model mice (left), as well as the analysis in the MPTP/p model mice (right) (*n* = 4 animals per condition). Samples were collected after 7 days of last MPTP injection. Data is presented as mean ± SEM, Student’s *T* test, **p < 0.01, *** *p* < 0.001 versus the corresponding saline group

Our previous study demonstrated that NLRP3 inflammasome-mediated neuroinflammation aggravates the process of PD [14]. NLRP1, NLRP2, NLRP3, NLRC4, and AIM2 inflammasomes within the CNS have generated the most attention in neuroscience [22]. MPTP is a commonly used toxin when establishing the PD model for research in vivo, so we prepared an acute MPTP and a chronic MPTP/p model in order to detect the changes in the expressions of NLRP1, NLRP2, NLRP3, NLRC4, and AIM2 in the mouse midbrain. As shown in Fig. 1a, the expressions of NLRP1 and NLRP3 were upregulated, not only in acute MPTP model, but also in chronic MPTP/p model, without changes in other inflammasomes (*T* test, *p* < 0.001). These results suggested that the NLRP1 and the NLRP3 inflammasome activation could be involved in the pathogenesis of PD.

Systemic inflammation is accompanied and aggravated by the pathological process of PD [23], so the anti-inflammatory agents have become a novel therapeutic focus. The liver is the main peripheral organ involved in metabolism and in inflammation. We detected the expression of inflammasomes in the liver under MPTP challenge. The acute MPTP model generally triggered inflammatory response. The expressions of NLRP2, NLRP3, and NLRC4 were remarkably higher in the liver of MPTP group (*T* test, *p* < 0.001) (Fig. 1c). The chronic MPTP/p model demonstrated upregulated expressions of NLRP2 and NLRP3 (*T* test, *p* < 0.01) (Fig. 1d, right).

Macrophages could be transferred through the blood brain barrier to change into microglia, playing a dual role in regulating the immune and inflammatory responses in both the periphery and the CNS [12]. Bone marrow-derived macrophages (BMDM) are typical representative of macrophages. We cultured the BMDM from two types of MPTP model mice in order to evaluate the expressions of the inflammasomes. Figure 1e shows that both NLRP2 and NLRP3 expressions increased in the BMDM of MPTP-treated mice (*T* test, *p* < 0.001). The NLRP3 expression exhibited the most common change in the midbrain, the liver, and BMDM in response to MPTP insult. These results suggested that NLRP3 inflammasome activation could exert a crucial role in the development of PD.

### Reduction of NLRP3 in the liver by in vivo siNlrp3 wrapped with lentivirus via the tail vein administration

**Fig. 2:**
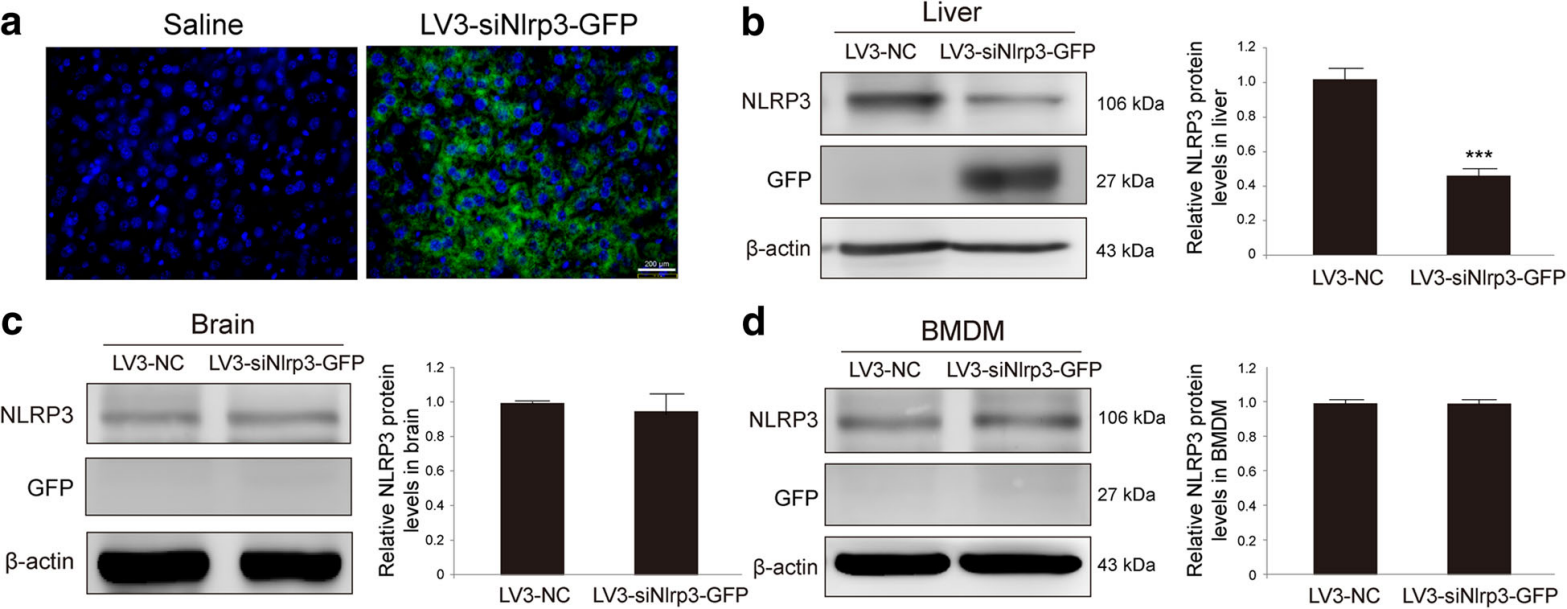
The expression of NLRP3 protein was decreased in the liver by siRNA administration in vivo without effects on brain or BMDM. **a** Immunofluorescence staining for GFP in the liver with × 10 objective, bar 200 μm. **b** Analysis of the NLRP3 and the GFP expression in liver samples from the siRNA negative control with the lentivirus (LV3-NC) or the *Nlrp3* siRNA with the GFP (LV3-siNlrp3-GFP) mice via the tail vein. **c** Analysis of NLRP3 and GFP expression in the midbrain samples from the LV3-NC or the LV3-siNlrp3-GFP mice via the tail vein. **d** Analysis of NLRP3 and GFP expression in the BMDM samples from the LV3-NC or the LV3-siNlrp3-GFP mice via the tail vein (*n* = 4 animals per condition). Samples were collected after 7 days of last saline injection. Data is presented as mean ± SEM, Student’s *T* test, ***p < 0.001 versus the LV3-NC group

To further determine if NLRP3 inflammasome played a critical role in the inflammatory response in PD, the siNlrp3 wrapped with lentivirus (LV3-siNlrp3) was intravenous injected into the mouse tail. The siNlrp3 wrapped with lentivirus (LV3-siNlrp3) showed obvious green fluorescent in the liver sections (Fig. 2a) and NLRP3 expression was decreased by 56.4% (Fig. 2b) after injecting green fluorescent protein (GFP)-marked LV3-siNlrp3 (*T* test, *p* < 0.001). The LV3-siNlrp3 injections had no effects on NLRP3 protein expression in the midbrain tissue or the BMDM lysates (Fig. 2c–d). The data indicated that the LV3-siNlrp3 predominantly inhibited hepatic NLRP3 protein expression via the tail vein injection. The downregulated NLRP3 expression did not occur in the brain nor in the BMDM at the current experimental conditions.

### Reduction of NLRP3 expression has no effects on liver morphology and MPTP metabolism

**Fig. 3:**
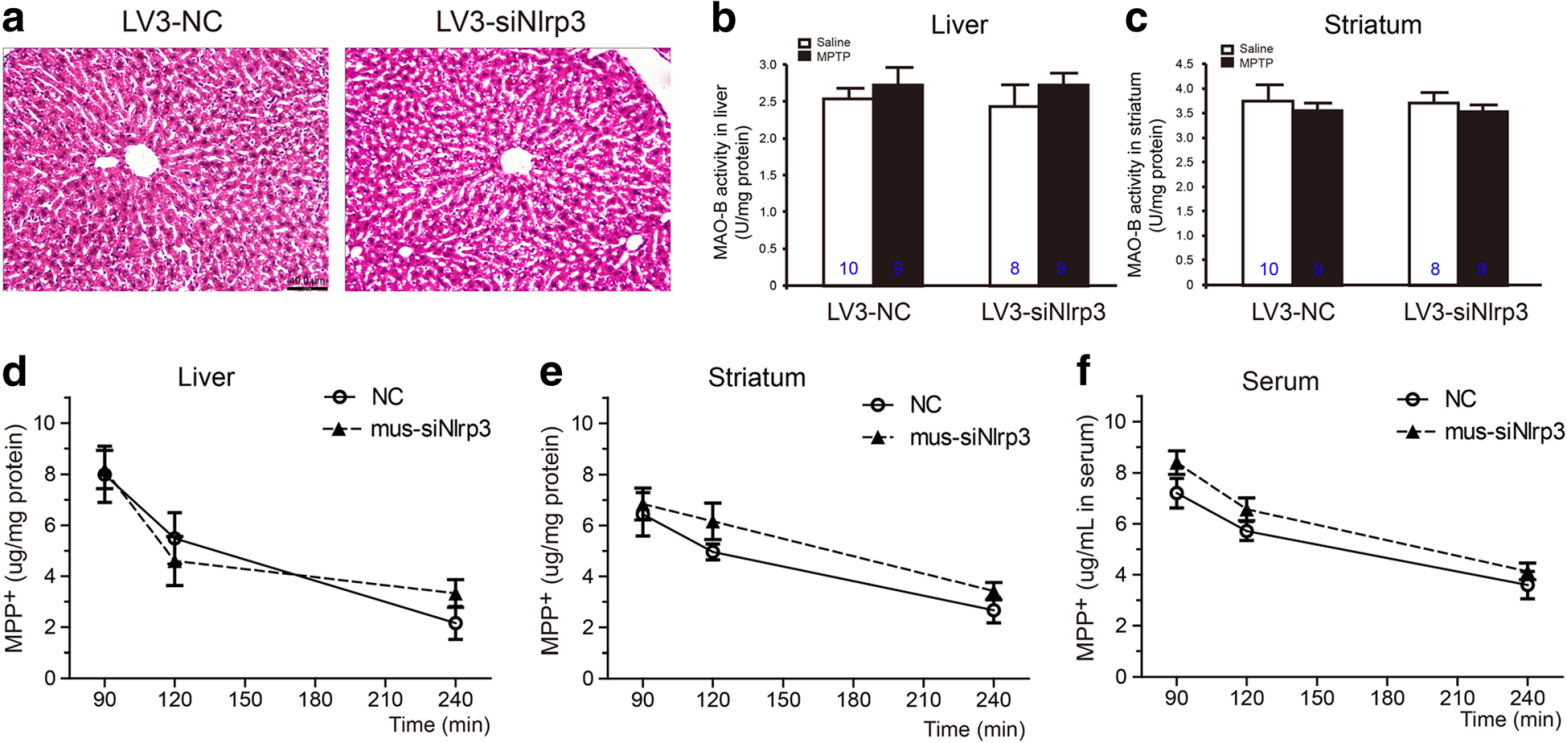
Silencing of *Nlrp3* in the liver by siRNA administration in vivo has no effects on the metabolism of MPTP. **a** Representative images from hematoxylin and eosin staining in the liver samples. **b**, **c** The analysis of MAO-B activity in the liver and the striatum via ELISA (*n* = 4 animals per condition). Samples were collected after 7 days of last MPTP injection. Data is presented as mean ± SEM, two-way ANOVA. **d**, **f** MPP^+^ levels were determined at the times indicated following the treatment with MPTP in liver, striatum, and serum (*n* = 8 animals per condition). Samples were collected after 90, 120, and 240 min of MPTP single injection. Data is presented as mean ± SEM, two-way ANOVA

The LV3-siNrp3 could significantly inhibit the expression of NLRP3 protein, so hematoxylin and eosin staining were examined the histopathology of the livers. The siRNA-injected mice had no liver damage or inflammatory cell infiltrates surrounding the portal or the central veins (Fig. 3a). The MPTP was converted by MAO-B to its toxic metabolite MPP^+^ (1-methyl-4-phenylpyridinium ion) via the MPDP^+^ (1-methyl-4-phenyl-2, 3-dihydropyridinium ion). Both the parent compound and major metabolite MPP^+^ were toxic to hepatocytes and brain cells [24]. We then determined if the LV3-siNrp3 affected the MPTP metabolism mediated by MAO-B in the liver and the striatum. Our results showed that neither the liver (two-way ANOVA, treatment: F_1,12_ = 0.469, *p* = 0.506;siRNA: F_1,12_ = 0.925, *p* = 0.335; interaction: F_1,12_ = 3.491, *p* = 0.086) (Fig. 3b) nor the striatum exhibited an altered (two-way ANOVA, treatment: F_1,12_ = 0.775, *p* = 0.396; siRNA: F_1,12_ = 0.125, *p* = 0.396; interaction: F_1,12_ = 1.026, *p* = 0.331) (Fig. 3c) activity of MAO-B after LV3-siNrp3 injection or MPTP treatment.

We further investigated the metabolism of MPTP transformed into MPP^+^ that was transported to dopaminergic (DA) neurons via the transporter into the neuronal bodies and neuritis, thus inhibiting mitochondrial complex I activity. Following the MPTP injection after 90, 120, or 240 min, the mice were euthanized, and the serum, the liver, and the striatum were acquired for MPP^+^ detection via HPLC. As shown in Fig. 3d, the concentration of MPP^+^ in the liver decreased with time in both the LV3-NC- and the LV3-siNlrp3-injected mice, but showed no difference between these two groups. The same variation tendency was observed in the detections of the striatum and the serum (Fig. 3e–f). These results indicated that the downregulation of NLRP3 in the liver had no effect on hepatic morphology and conversion of MPTP into active MPP^+^, which maintained the biological activity of the MPTP administration.

### Reduction of NLRP3 in the liver by in vivo siRNA administration reduces MPTP-induced inflammatory response

**Fig. 4:**
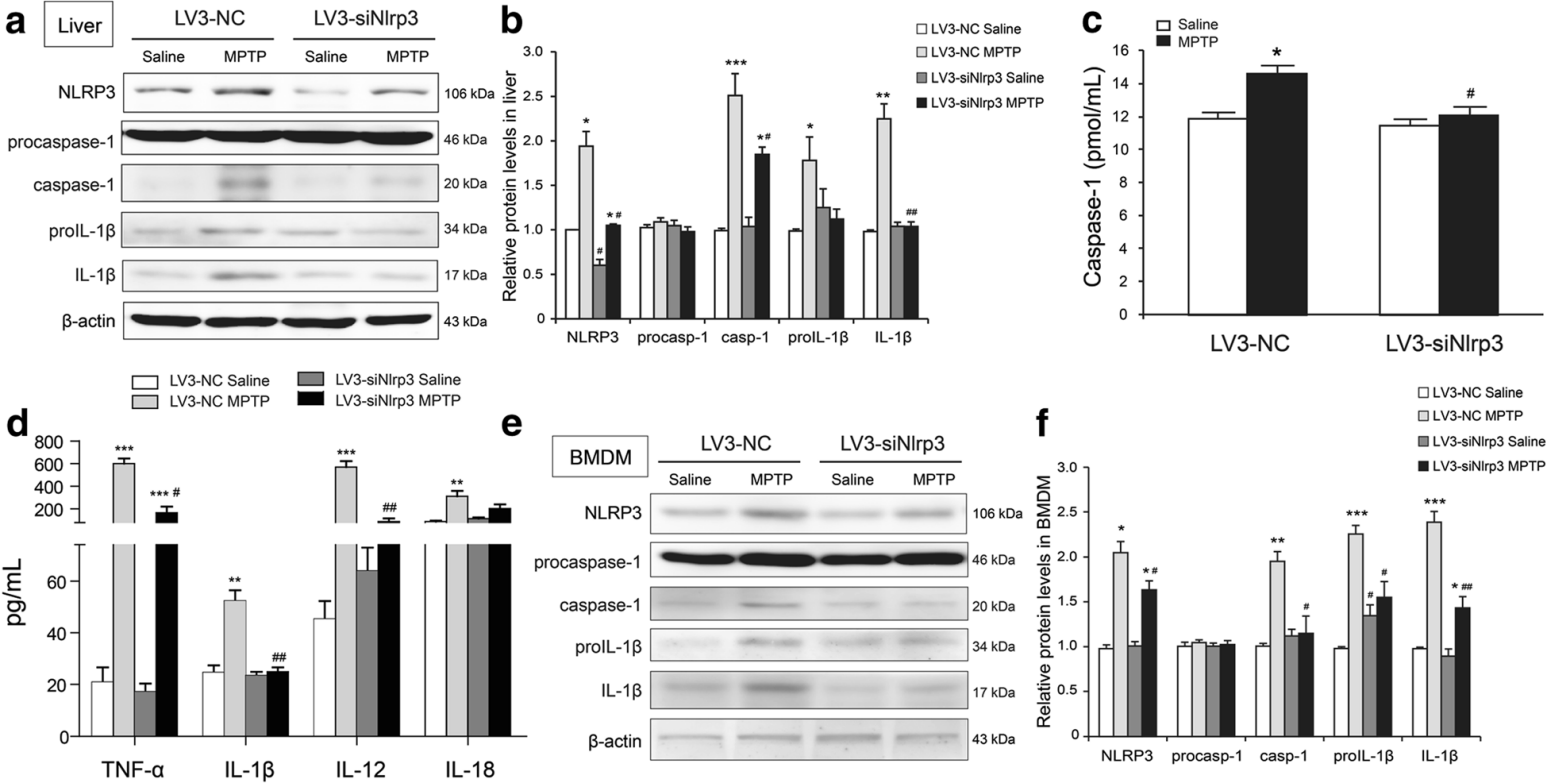
MPTP-induced inflammation was reduced in the serum, liver, and BMDM from mice injected with LV3-siNlrp3. **a** Blots of NLRP3 activation signaling markers in the liver samples from the LV3-NC or the LV3-siNlrp3 mice 7 days post MPTP treatment. **b** Analysis with the indicated antibodies (*n* = 4 animals per condition). Data is presented as mean ± SEM, two-way ANOVA, *p < 0.05, **p < 0.01, ***p < 0.001 versus the corresponding saline group; ^#^*p* < 0.05, ^##^*p* < 0.01 versus corresponding LV3-NC group. **c**, **d** The secretion of caspase-1, TNF-α, IL-β, IL-12, and IL-18 to serum were determined via ELISA (*n* = 8 animals per condition). Data is presented as mean ± SEM, two-way ANOVA, **p < 0.01, ***p < 0.001 versus the corresponding saline group; ^#^*p* < 0.05, ^##^*p* < 0.01 versus the corresponding LV3-NC group). **e**, **f** Representative blots and analysis of NLRP3 activation signaling makers in the BMDM samples from the LV3-NC or the LV3-siNlrp3 mice 7 days post MPTP treatment (*n* = 4 animals per condition). Samples were collected after 7 days of last MPTP injection. Data is presented as mean ± SEM, two-way ANOVA,*p < 0.05, **p < 0.01, ***p < 0.001 versus the corresponding saline group; ^#^*p* < 0.05, ^##^*p* < 0.01 versus the corresponding LV3-NC group

The secretion of pro-inflammatory IL-1β and caspase-1 are typical features of NLRP3 inflammasome activation [17]. We examined the activation of NLRP3 inflammasome in the liver after acute MPTP treatment and injection with LV3-siRNA since the liver is rich in NLRP3 protein expression. The NLRP3 protein expression increased by the MPTP injury in the liver, although the rising trend was stopped by the LV3-siNlrp3 injection (two-way ANOVA, treatment: F_1,12_ = 28.925, *p* = 0.008; siRNA: F_1,12_ = 17.664, *p* = 0.010; interaction: F_1,12_ = 35.025, *p* < 0.001) (Fig. 4a). The MPTP increased the expressions of IL-1β (two-way ANOVA, treatment: F_1,12_ = 9.771, *p* = 0.030; siRNA: F_1,12_ = 19.617, *p* = 0.005; interaction: F_1,12_ = 8.287, *p* = 0.010) and caspase-1 (two-way ANOVA, treatment: F_1,12_ = 10.236, *p* = 0.005; siRNA: F_1,12_ = 11.307, *p* = 0.017; interaction: F_1,12_ = 6.899, *p* = 0.043) in the liver when injected with LV3-NC more than the saline treatment. We analyzed the expressions of IL-1β and caspase-1, where in accordance with the NLRP3 downregulation in the livers, the MPTP failed to induce a dramatic elevation in IL-1β and caspase-1 expression in the LV3-siNlrp3-injected mice (Fig. 4a–b). Silencing the *Nlrp3* in the liver via in vivo siRNA administration could control the activation of NLRP3 inflammasome.

Liver damage could deteriorate the secretion of the pro-inflammatory cytokines in serum and the regulation of inflammation levels in surrounding cells, including the inflammatory-sensitive BMDM. We found that the activation products of inflammasomes, caspase-1 and IL-1β, increased in the serum of MPTP-treated mice following the LV3-NC injection (Fig. 4c–d). The siNlrp3 reduced or even abolished the secretion of caspase-1 (two-way ANOVA, treatment: F_1,12_ = 11.665, *p* = 0.005; siRNA: F_1,12_ = 8.968, *p* = 0.011; interaction: F_1,12_ = 5.033, *p* = 0.045), IL-1β (two-way ANOVA, treatment: F_1,12_ = 29.265, *p* < 0.001; siRNA: F_1,12_ = 27.845, *p* < 0.001; interaction: F_1,12_ = 23.815, *p* < 0.001), and IL-18 (two-way ANOVA, treatment: F_1,12_ = 14.039, *p* = 0.003;siRNA: F_1,12_ = 1.789, *p* = 0.206; interaction: F_1,12_ = 4.587, *p* = 0.053) following the MPTP administration. The siNlrp3 treatment had a mild effect on serum TNF-α (two-way ANOVA, treatment: F_1,12_ = 103.161, *p* < 0.001; siRNA: F_1,12_ = 37.423, *p* < 0.001; interaction: F_1,12_ = 36.167, *p* < 0.001) and IL-12 (two-way ANOVA, treatment: F_1,12_ = 80.722, *p* = 0.000; siRNA: F_1,12_ = 57.194, *p* < 0.001; interaction: F_1,12_ = 66.859, *p* < 0.001) production, which was inflammasome-independent (Fig. 4d). We further investigated the role LV3-siNlrp3 by siRNA administration via the tail vein in vivo had in the inflammatory-sensitive BMDM cells*.* As shown in Fig. 4e, the siNlrp3 injection via the tail vein did not change the expression of NLRP3 protein in BMDM, but the MPTP significantly increased the NLRP3 expression in the BMDM of both the siRNA-treated mice (two-way ANOVA, treatment: F_1,12_ = 12.057, *p* = 0.012; siRNA: F_1,12_ = 5.881, *p* = 0.052; interaction: F_1,12_ = 9.701, *p* = 0.041). The MPTP accelerated the expression of pro-IL-1β (two-way ANOVA, treatment: F_1,12_ = 22.059, *p* < 0.001; siRNA: F_1,12_ = 13.569, *p* = 0.036; interaction: F_1,12_ = 7.996, *p* = 0.035), IL-1β (two-way ANOVA, treatment: F_1,12_ = 11.665, *p* = 0.005; siRNA: F_1,12_ = 8.968, *p* = 0.011; interaction: F_1,12_ = 5.033, *p* = 0.045), and caspase-1 (two-way ANOVA, treatment: F_1,12_ = 31.208, *p* = 0.002; siRNA: F_1,12_ = 7.533, *p* = 0.045; interaction: F_1,12_ = 13.609, *p* = 0.041) in the BMDM of the LV3-NC-injected mice, although the siNlrp3 lessened the activation of NLRP3 inflammasome (Fig. 4e–f). These results indicated that the siNlrp3 injected into the tail vein could inhibit MPTP-induced inflammation via suppressing NLRP3 inflammasome activation.

### Nlrp3 siRNA injected via tail vein prevents MPTP-induced NLRP3 inflammasome activation in the brain and rescues the TH neuron loss

**Fig. 5:**
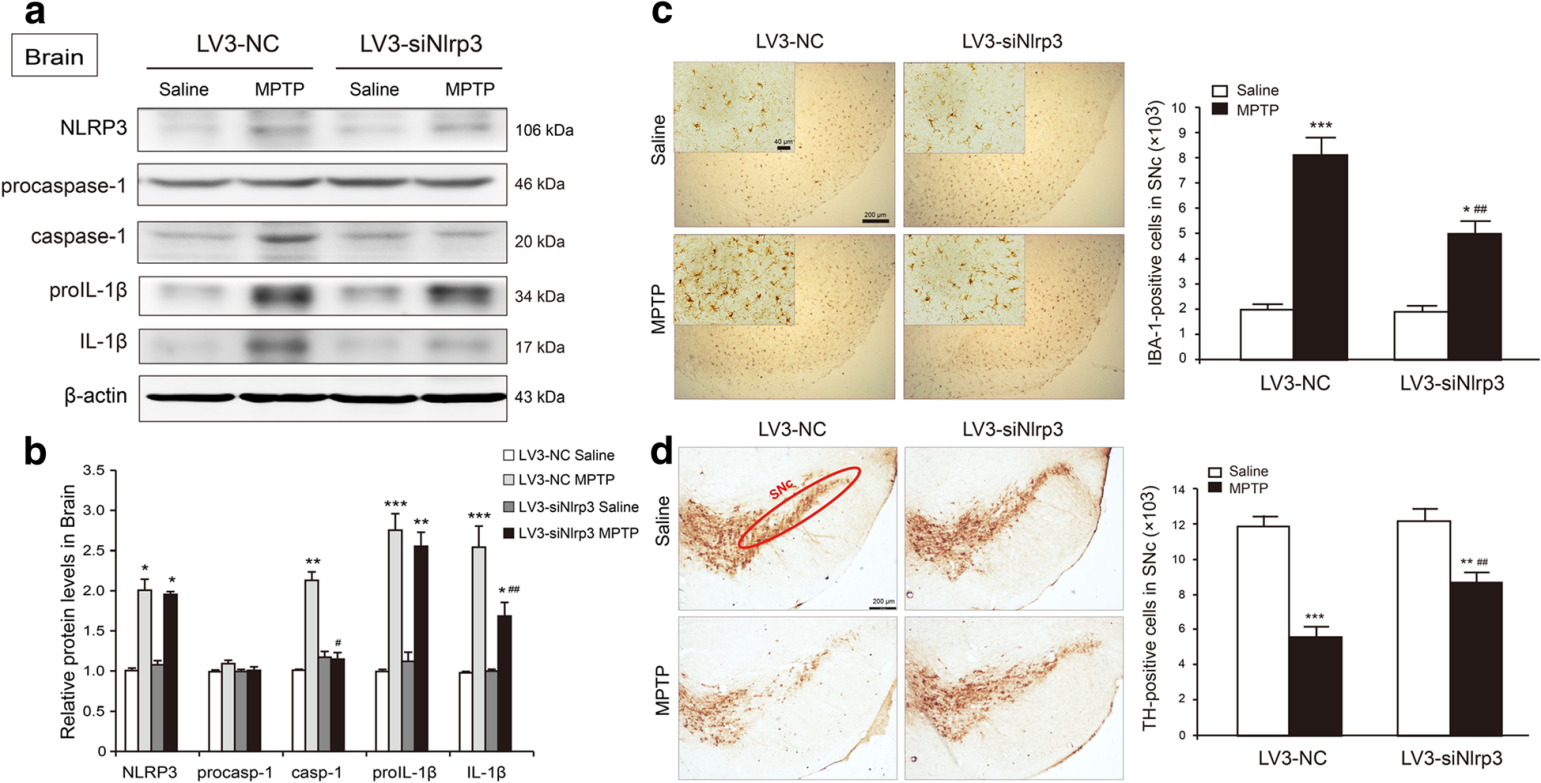
Reduction of *Nlrp3* decreased MPTP-induced NLRP3 inflammasome activation and TH neuronal loss in SNc from mice injected with LV3-siNlrp3 via the tail vein. **a**, **b** Representative blots and analysis of NLRP3 activation signaling makers in the midbrain samples from the LV3-NC or the LV3-siNlrp3 mice 7 days post MPTP treatment. **c** Representative images (left) and quantitative analyses (right) of the positive cell numbers showing immunochemical label microglia using the IBA-1 in the SNc of mice injected with the LV3-NC or the LV3-siNlrp3 7 days post MPTP treatment. **d** Representative images (left) and quantitative analyses (right) of positive cell numbers showing immunochemical label DA neurons using TH in the SNc of mice injected with the LV3-NC or the LV3-siNlrp3 7 days post MPTP treatment (*n* = 4 animals per condition). Samples were collected after 7 days of last MPTP injection. Data is presented as mean ± SEM, two-way ANOVA,*p < 0.05, **p < 0.01, ***p < 0.001 versus corresponding the saline group; ^#^*p* < 0.05, ^##^*p* < 0.01 versus the corresponding LV3-NC group

Systemic inflammation accelerates the production of pro-inflammatory cytokines in the brain and the pathological process of PD [17]. To determine if the tail vein injection of siNlrp3 inhibited the MPTP-induced inflammation in the brain by controlling the NLRP3 inflammasome activation, the mice were treated with MPTP after siRNA injection. The results showed that MPTP-induced additional NLRP3 expression in the midbrain of the LV3-NC-injected mice, but there was no change in the LV3-siNlrp3-injected mice (two-way ANOVA, treatment: F_1,12_ = 19.205, *p* = 0.012; siRNA: F_1,12_ = 6.651, *p* = 0.052; interaction: F_1,12_ = 16.609, *p* = 0.037) (Fig. 5a). The MPTP increased the expression of pro-IL-1β (two-way ANOVA, treatment: F_1,12_ = 29.205, *p* < 0.001; siRNA: F_1,12_ = 7.071, *p* = 0.064; interaction: F_1,12_ = 5.992, *p* = 0.083), IL-1β (two-way ANOVA, treatment: F_1,12_ = 24.088, *p* = 0.002; siRNA: F_1,12_ = 9.139, *p* = 0.032; interaction: F_1,12_ = 12.609, *p* = 0.003), and caspase-1 (two-way ANOVA, treatment: F_1,12_ = 16.594, *p* = 0.009;siRNA: F_1,12_ = 8.301, *p* = 0.058; interaction: F_1,12_ = 12.929, *p* = 0.041) in the midbrain of the LV3-NC-injected mice. The siNlrp3 decreased the MPTP-induced IL-1β and caspase-1 expression but had little effect on pro-IL-1β expression, which suggested that the siNlrp3 injected via tail vein only reduced the cleavage and the maturation of pro-IL-1β, rather than its production (Fig. 5a, b).

Microglia is the main immune cell and regulates the cytokines production in the CNS. The activation and the proliferation of microglia further aggravate the inflammatory response [25, 26]. We determined if the tail vein injection of siNlrp3 could influence the MPTP-induced microglia activation in the CNS. As shown in Fig. 5c, the siRNA injected via tail vein had no effect on the activation or the proliferation of microglia at the basal line. The MPTP injury accelerated the swelling and the proliferation of microglia, where it elongated the neuritis of microglia in the SNc of the LV3-NC-injected mice. The siNlrp3 prominently decreased the MPTP-induced activation and proliferation of the microglia in the SNc (two-way ANOVA, treatment: F_1,12_ = 187.810, *p* = 0.000; siRNA: F_1,12_ = 19.263, *p* = 0.001; interaction: F_1,12_ = 21.537, *p* = 0.001) (Fig. 5c). The change of brain microenvironment and microglia activation induced dopaminergic neuronal injury [21, 27]. We used TH antibody-labeled DA neurons via immunohistochemistry, where the siRNA injected via tail vein had no effects on DA neurons at the basal line. The MPTP resulted in the TH^+^ neurons decreasing by nearly 53.5% (two-way ANOVA, treatment: F_1,12_ = 53.979, *p* < 0.001; siRNA: F_1,12_ = 6.610, *p* = 0.025; interaction: F_1,12_ = 4.444, *p* = 0.057) (Fig. 5d). The MPTP-induced TH neuronal loss was alleviated by the siNlrp3 injected via tail vein, evidenced by 20.8% loss of TH^+^ neurons. These results revealed that the inhibition of hepatic NLRP3 inflammasome prevented MPTP-induced neuroinflammation and DA neuronal degeneration in the mouse midbrain.

## Discussion

Elevated levels of inflammatory cytokines in the brain, peripheral organs, cerebral spinal fluid (CSF), and serum of PD patients support the existence of functional interconnections between the immune and nervous systems [28]. Several reports showed that DA neuronal loss in PD originates from neuroinflammation and is triggered by systemic circulating inflammatory molecules [10, 28]. Furthermore, innate immunity is the first line of defense in infection, which plays a vital role in tissue repair, clearance of apoptotic cells, and cellular debris [29]. Within innate immunity, inflammasomes act as an important immune defense in central and peripheral tissues [30]. The production and the maturation of IL-1β/IL-18 were controlled by the activation of NLRP inflammasomes [31, 32]. Our previous study showed that the microglial NLRP3 inflammasome activation is responsible for the DA neuronal degeneration in a MPTP-induced PD mouse model [14]. The roles that the liver inflammasome play in neurological damage of PD remain unknown.

It has been considered that the liver is the first line of defense against MPTP. Damage to other tissues may be secondary to the liver in response to MPTP challenge. MPTP toxicity is mainly due to inhibition of complex I in the mitochondrial electron transport chain, and the generation of ROS. ROS can induce a series of inflammatory events, which subsequently aggravates ROS generation. Excessive ROS in the liver can exacerbate the inflammatory responses by activating several pro-inflammatory signaling, such as NF-κB, MAPK, and JAK-STAT pathways. Consequently, nuclear NF-κB p65 subunit will activate signal 1 of NLRP3 inflammasome and upregulate the expression of NLRP3. Therefore, ROS accumulation contributes to MPTP-induced NLRP3 upregulation in the liver. In the present study, we attempt to confirm that further changes in the brain are the consequence of the levels of hepatic inflammation, without impact on the levels of MPP^+^ and MAO-B activity in the brain.

Growing evidence indicates that systemic inflammation aggravates the progress of PD, and inhibiting inflammatory cytokine production or blocking cytokine receptor can alleviate DA neuron damage in multiple animal models of PD [33]. However, the ability to alleviate the inflammatory molecules from peripheral tissues to CNS remains unknown. The liver is the main metabolic organ in the periphery and a large number of inflammatory cytokines originate from the mammal liver, so we analyzed the MPTP-induced inflammatory response in the liver for a pointcut to mimic systemic inflammation. An amount of inflammasomes (e.g., NLRP1, NLRP2, NLRP3, NLRC4, AIM2) was present in both the CNS and the peripheral tissues [34]. In the present study, we clarified that the amplitude of change in NLRP3 is the most remarkable whether in the midbrain, the liver, or the BMDM in the acute and the chronic MPTP model. Therefore, we speculated that NLRP3 inflammasome may be the most sensitive to MPTP challenge among NLRP family members.

We then used the LV3-siNlrp3 to downregulate the expression of hepatic NLRP3 protein via tail vein injection. The MPTP acute model of PD was established 7 days later. The siNlrp3 could significantly decrease the NLRP3 expression in the liver, although it had almost no impact in other organs, such as the brain. The results found that the reduction of NLRP3 in the liver could decrease the release of pro-inflammatory cytokines from the liver into serum and brain, even inhibiting MPTP-induced microglia activation and DA neuron loss in the mouse SNc, without affecting the metabolism of MPTP. These findings suggested that the NLRP3 in the liver mediated the immune signaling and played an unexpected role in the central nerve injury of PD. The downregulation of the NLRP3 expression in the liver attenuated MPTP-triggered systemic inflammation and inhibited the activation of the microglia and the loss of the DA neurons in the midbrain. This study demonstrated that the inhibition of the NLRP3 inflammasome activation in the liver could alleviate the MPTP-induced neural injury, which could provide novel target for modulating systemic inflammation in the pathogenesis of PD. Compared with traditional anti-inflammatory drugs, targeting NLRP3 can selectively inhibit IL-1β/IL-18 production without impact on some other beneficial cytokines, such as IL-4 and IL-10. Therefore, NLRP3 inhibitors may have the characteristics of the higher selectivity and the fewer side effects.

The predominant innate immune cells in the brain are microglia, although macrophages and astrocytes also contribute to the innate immune reposes in the CNS [35]. BMDM is a common macrophage that assumes the regulation of immune and inflammation in the periphery [36]. The BMDM-released pro-inflammatory cytokines (e.g., TNF-α, IL-1β) and even the BMDMs themselves could transfer from the BBB into the brain, which in turn transformed into microglia, where it played a crucial role of immunoregulation [37]. The downregulation of NLRP3 in the liver did not change the expression of NLRP3 protein in the BMDM but did decrease the release of pro-inflammatory factors from the BMDM, such as IL-1β and caspase-1. We speculate that NLRP3-targeted siRNA does not reach the bone marrow, and the changes in other inflammatory markers in BMDM might be due to general levels of liver inflammation. Therefore, the siNlrp3 injection may secondarily suppress the MPTP-induced NLRP3 inflammasome activation, rather than downregulating the NLRP3 protein expression in the BMDMs. The results suggested that the tail vein injection of the siNlrp3 reduced the pro-inflammatory cytokines permeating the BBB into the brain. This meant that inhibition of the NLRP3 inflammasome in the liver contributed to alleviating inflammatory molecules spreading into the brain and delayed the progress of MPTP-induced neuroinflammation and DA neuron damage.

At last, MPTP-induced animal models of PD merely show an acute, severe, and pure dopaminergic deficiency and display a homogeneous behavioral disturbance. Meanwhile, cognitive, emotional, and other nondopaminergic signs are difficult to evaluate in MPTP-induced animal model. Furthermore, there are significant differences in species between humans and animals; thus, pharmacological effective drugs in MPTP animal models always have no curative effect in clinical application. Therefore, we need to further develop clinical trials to get patient data support and clarify the exact key molecules and mechanisms involved in inflammation from the periphery to the brain in the pathogenesis of PD.

## Conclusions

The present study used tail vein injection of the LV3-siNlrp3 to explore the potential influence of hepatic NLRP3 inflammasome-mediated inflammation on neuronal injury in the MPTP-induced PD model. Our findings indicated that the inhibition of hepatic NLRP3 inflammasome activation protected DA neurons against MPTP-induced systemic inflammation. This study provided insights into the direct linkage between liver inflammation and neuroinflammation in the pathogenesis of PD and provided the potential target of NLRP3, in terms of opening up a novel avenue for developing PD therapeutic drugs.

## Additional file

Additional file 1 **Figure S1.** Brilliant bright blue alleviated MPP^+^-induced cell apoptosis and activation of inflammation in SH-SY5Y cells. (A) Cells were stained with Hoechst 33342 and observed by fluorescence microscopy. Representative pictures are presented. (B) Cell viability was measured by CCK-8 assay. (C) Representative immunoblots and quantification for analysis of NLRP3, pro-caspase 1, and pro-IL-1β in cell lysates. (D) Caspase 1 and IL-1β in cell culture supernatants. Data are represented as mean ± SEM from three independent experiments. **P < 0.01, ***P < 0.001 vs. CTL group, ^#^*P* < 0.05, ^##^*P* < 0.01, ^###^*P* < 0.001 vs. MPP^+^-treated group. (DOCX 108 kb)

## Abbreviations

BBB: Blood brain barrier
BMDM: Bone marrow-derived macrophages
BSA: Bovine serum albumin
CNS: Central nervous system
CSF: Cerebral spinal fluid
DA: Dopaminergic
GFP: Green fluorescent protein
GM-CSF: Granulocyte-macrophage colony stimulating factor
HE: Hematoxylin and eosin
HPLC: High-performance liquid chromatography
IBA-1: Ionized calcium binding adaptor molecule 1
IL-1β: Interleukin-1β
MAO-B: Monoamine oxidase B
MPTP: 1-Methyl-4-phenyl-1, 2, 3, 6-tetrahydropyridine
NLRP: Nod-like receptor protein
PBS: Phosphate-buffered saline
PD: Parkinson’s disease
SNc: Substantia nigra pars compacta
TH: Tyrosine hydroxylase
