# Supplementary material for: Inhibition of the hepatic Nlrp3 protects dopaminergic neurons via attenuating systemic inflammation in a MPTP/p mouse model of Parkinson’s disease
Source: J Neuroinflammation. 2018 Jul 2;15:193. doi: 10.1186/s12974-018-1236-z (PMC6029067; doi:10.1186/s12974-018-1236-z)
Supplement: Supplementary file 1 — Figure S1. Brilliant bright blue alleviated MPP+-induced cell apoptosis and activation of inflammation in SH-SY5Y cells. (A) Cells were stained with Hoechst 33342 and observed by fluorescence microscopy. Representative pictures are presented. (B) Cell viability was measured by CCK-8 assay. (C) Representative immunoblots and quantification for analysis of NLRP3, pro-caspase 1, and pro-IL-1β in cell lysates. (D) Caspase 1 and IL-1β in cell culture supernatants. Data are represented as mean ± SEM from three independent experiments. **P < 0.01, ***P < 0.001 vs. CTL group, #P < 0.05, ##P < 0.01, ###P < 0.001 vs. MPP+-treated group. (DOCX 108 kb) [file 12974_2018_1236_MOESM1_ESM.docx]

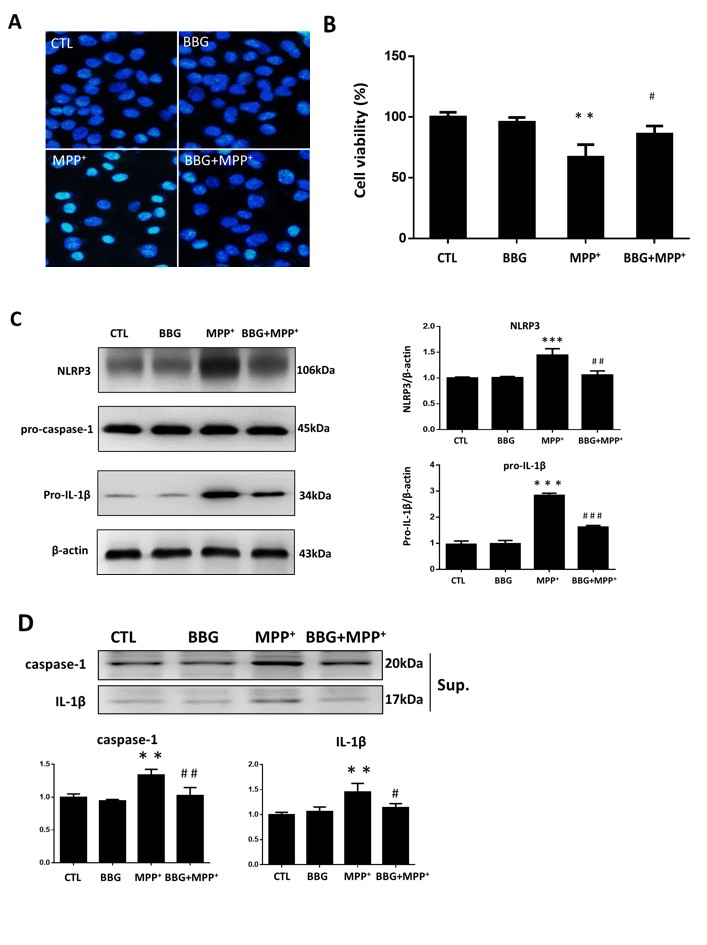


**Figure S1. Brilliant bright blue alleviated MPP^+^-induced cell apoptosis and activation of inflammation in SH-SY5Y cells.** (A) Cells were stained with Hoechst 33342 and observed by fluorescence microscopy. Representative pictures are presented. (B) Cell viability was measured by CCK-8 assay. (C) Representative immunoblots and quantification for analysis of NLRP3, pro-caspase 1 and pro-IL-1β in cell lysates. (D) Caspase 1 and IL-1β in cell-culture supernatants. Data are represented as mean ± SEM from three independent experiments. ***P* < 0.01, ****P* < 0.001 *vs*. CTL group, ^#^*P* < 0.05, ^##^*P* < 0.01, ^###^*P* < 0.001 *vs*. MPP^+^-treated group.
